# Empowering Foot Care Literacy Among People Living With Diabetes and Their Carers With an mHealth App: Protocol for a Feasibility Study

**DOI:** 10.2196/52036

**Published:** 2023-11-21

**Authors:** Huiling Liew, Anita Pienkowska, Chin-Siang Ang, Muhammad Daniel Azlan Mahadzir, Kelley Fann Ing Goh, Nandika Lodh, Iva Bojic, Ashwini Lawate, Qi Chwen Ong, Kavita Venkataraman, Josip Car, Andy Hau Yan Ho

**Affiliations:** 1 Department of Endocrinology Tan Tock Seng Hospital Singapore Singapore; 2 Lee Kong Chian School of Medicine Nanyang Technological University Singapore Singapore; 3 Saw Swee Hock School of Public Health National University of Singapore Singapore Singapore

**Keywords:** digital therapeutics, mHealth, diabetes mellitus, diabetes foot ulcer, feasibility study, mobile phone

## Abstract

**Background:**

Diabetic foot ulcers (DFUs) cause significant morbidity affecting 19% to 34% of people living with diabetes mellitus. DFUs not only impair quality of life but may also result in limb loss and mortality. Patient education has been advocated to raise awareness of proper foot self-care and the necessity of seeking assistance when a foot wound occurs. Modern technologies, including mobile health (mHealth) interventions such as health apps, bring the potential for more cost-effective and scalable interventions.

**Objective:**

This study aims to examine the feasibility and usability of a newly developed mHealth app called Well Feet, which is a diabetes and foot care education app for individuals at risk of developing DFU.

**Methods:**

Well Feet was developed using an evidence-based and expert panel cocreation approach to deliver educational content available in 3 languages (ie, English, Chinese, and Malay) via animation videos and a range of additional features, including adaptive learning. A nonrandomized, single-arm feasibility study using a mixed methods approach with a series of validated questionnaires and focus group discussions will be conducted. In total, 40 patients and carers will be recruited from a tertiary hospital diabetes clinic to receive a 1-month mHealth intervention. The primary outcomes are the usability of the app and a qualitative perspective on user experience. Secondary outcomes include changes in foot care knowledge, self-management behaviors, and quality of life.

**Results:**

Patient recruitment began in July 2023, and the intervention and data collection will be completed by the end of September 2023. This study has been approved by National Healthcare Group Domain Specific Review Board (2022/00614) on February 10, 2023. The expected results will be published in spring 2024.

**Conclusions:**

Through this feasibility study, the Well Feet DFU education app will undergo a comprehensive quantitative and qualitative evaluation of its usability and acceptance for future improvement in its design. With local contextualization, cultural adaptation, and its multilingual functionality, the app addresses a critical aspect of DFU health education and self-management in a multiethnic population. Findings from this study will refine and enhance the features of the app based on user feedback and shape the procedural framework for a subsequent randomized controlled trial to assess the effectiveness of Well Feet.

**Trial Registration:**

ClinicalTrials.gov NCT05564728; https://clinicaltrials.gov/study/NCT05564728

**International Registered Report Identifier (IRRID):**

DERR1-10.2196/52036

## Introduction

### Background

According to the International Diabetes Federation, 537 million people worldwide (ie, 1 in 10) lived with diabetes in 2021, and this number is expected to rise to 783 million by 2045 [1]. The lifetime incidence of developing a diabetic foot ulcer (DFU) in adults living with diabetes ranges from 19% to 34% [2]. In a 2017 systematic review and meta-analysis, DFU's global prevalence among people with diabetes was estimated to be 6.3%, with Asia having a prevalence of 5.5% [3]. Diabetes-related lower extremity complications affected an estimated 131 million people (1.8% of the total global population) in 2016, with overall age-standardized rates for years lived with disability increasing by 15.9% between 1990 and 2016 [4]. Southern sub-Saharan Africa, South Asia, and Southeast Asia experienced the greatest growth during the period. Despite multiple clinical [5], economic [6,7] and policy [8,9] attempts to address DFU in Singapore, the country is still struggling to combat the rising prevalence of DFU-related complications with a reported 1500 DFU-related lower-limb amputations performed each year [10]. Furthermore, the estimated recurrence rate is 22.1% per person-year [11]. Hence, DFUs impose a significant clinical and economic burden on health care systems around the world as well as significant reductions in the quality of life for those affected [12].

Prevention initiatives, including lifelong patient education and continuous empowerment, are key strategies for successful foot care and management of diabetes, as recommended by evidence-based guidelines [13]. Whether delivered one-to-one or in small groups, diabetes foot health education is resource-intensive and costly—requiring the participation of doctors, nurses, podiatrists, diabetes educators, and allied health professionals. While valuable, foot care and diabetes education sessions lack immediacy, timeliness, access, and flexibility. In Singapore, 36% of patients with diabetes reported receiving no diabetes education [14] and over 51% spend less than a minute checking their feet [15]. Given the unmet need, financial and health workforce constraints, and being in line with modern approaches to education and self-management support, a digitally supported diabetes education mobile health (mHealth) apps using prevention and management of the DFU education program carries the potential to improve health care delivery and increase uptake, equity, and cost-effectiveness of health services related to DFU. Patient-facing technology such as mHealth apps can be blended with face-to-face care to augment the overall delivery of care and health services [16] and impact patients’ health outcomes [17].

The other agency that is heavily involved in the management of DFU is the carer. Informal carers are defined broadly as any family member, partner, or friend who provides support to a person having a chronic condition. Carers play a vital role particularly in assisting with wound care, monitoring changes, ensuring foot hygiene, managing medications, offering emotional support, advocating for medical care, and providing education [18]. Carer’s involvement improves patient outcomes, prevents complications, and enhances overall DFU care and well-being. For many people with chronic conditions, particularly the elderly, informal carers provide support and take responsibility for many of the care needs. Upskilling informal carers may result in better health outcomes [18]. Training equips them with skills to provide effective wound care, identify issues early, prevent complications, manage medications, offer emotional support [19] and promote timely access to health care. This improves the overall DFU management beyond the hospital walls, which may eventually result in reduction of hospitalizations, lowers costs of health care, and ensures collaborative, sustainable DFU management among patients, carers, and health care professionals.

### Related Work

Studies suggest that people living with diabetes show an interest in mHealth apps for preventing and monitoring DFUs [20]. There is a growing body of evidence supporting the potential of mHealth apps to enhance diabetes self-management performance [21]. A few studies have been conducted to assess or validate the potential of an mHealth app for diabetic foot care. Some of them focus on providing wound care trackers [22,23], others on empowering with knowledge on foot-ankle exercises [24], quick tips on foot care and first aid, reminders for medical check-ups, a contacts feature to save health providers’ contact information [25], text messaging [26], foot care essentials [27,28], foot care education in 6 animations [29] and tips for self-assessment [30]. Prior to a full trial, feasibility studies are routinely performed to determine whether a future study is feasible [31].

Some of the challenges identified in such interventions include maintaining patient engagement [25], tailoring content to the different stages of complexity of the disease, retaining and applying the knowledge acquired [32]. Thus, there is a need to explore the mechanisms by which evidence-based behavior change techniques and digital features can influence the uptake of digital diabetes and foot care education and ultimately improve diabetes health literacy and self-management behaviors. Even though the validation studies presented promising results, none of the mHealth apps for DFU comprise comprehensive in-depth education on all aspects of foot health, including prevention, inspection, and wound care while concurrently providing support for self-management with tracking features that focus both on feet health and general diabetes care. Additionally, no other tool addresses the need for a multilingual and adaptive solution that fits individual needs, such as the role (patient or caregiver), severity of the condition, and competency levels, concurrently reinforcing learning through notifications and spaced learning. Finally, there is a necessity to understand the user’s engagement and app effectiveness in detail using granular click-level data.

### Aim

This is a “proof-of-concept” feasibility study to evaluate a foot health education app for people living with diabetes, Well Feet, among patients and carers. Prior to a full trial, feasibility studies are routinely performed to determine whether a future study is feasible [31].

## Methods

### Study Design

This study is a nonrandomized, single-arm feasibility study with baseline, end point, and focus-group discussion design. An explanatory sequential mixed methods approach will be used to assess feasibility and usability of the mHealth app, Well Feet. Our study features a 1-month intervention period carefully designed to strike a balance between meaningful observation and participant convenience. This timeframe enables us to assess the feasibility of the intervention on our selected outcomes. All participants will receive Well Feet, and their feedback will be collected through quantitative and qualitative rating measures. Subsequently, focus group discussions will be conducted to provide valuable insights into their experiences. The reporting of this protocol follows CONSORT (Consolidated Standards of Reporting Trials) 2010 statement extension for feasibility studies [33]. This study has been registered at ClinicalTrials.gov (NCT05564728).

### Study Participants

#### Eligibility Criteria

Both patients and carers will be invited to participate in this study. Inclusion criteria for patients are (1) being diagnosed with type 2 diabetes and attending Tan Tock Seng Hospital Diabetes Clinic, (2) being 21 years or older, (3) being able to speak and read English, (4) owning a smartphone or tablet and can download the app, (5) having internet access, (6) being able to provide informed consent, (7) being Singaporeans or Singapore permanent residents. Inclusion criteria for carers are (1) carers who provided care for a patient with type 2 diabetes for the past 6 months, (2) being aged 21 years or older, (3) being able to speak and read in English, (4) owning a smartphone or tablet and being able to download the app, (5) having internet access, and (6) being able to provide informed consent. Exclusion criteria for patients and carers include being (1) pregnant, (2) an inpatient, or (3) having received formal training in medicine or allied health services.

#### Recruitment

Patients will be recruited through referrals from attending endocrinologists at Tan Tock Seng Hospital. Recruitment will take place during the patients’ routine clinic visits at Diabetes Clinic. The endocrinologists will screen for potential participants’ eligibility based on this study’s inclusion and exclusion criteria before referring them to the appointed research assistant to complete the recruitment process. At the same time, patients will be asked to nominate their carers to participate in this study. The nomination is optional and subject to patients’ and carers’ willingness. Notwithstanding, patients without a carer can still participate in this study. Potential patients and carers who choose not to participate in this study will be asked an open-ended question about the reasons. It will be highlighted that answering the question is voluntary. The information will be used to inform about the barriers to recruitment and acceptability of the intervention.

#### Informed Consent

The research assistant will conduct a briefing for all referred patients and carers in a closed space outside of the clinic room. The briefing involves explaining this study’s aim and study’s inclusion and exclusion criteria. Additionally, potential participants will be briefed thoroughly about this study’s procedures, risk exposure, and all the information included in the informed consent form (Multimedia Appendix 1). The research assistant will provide adequate time for the participants to decide. Participants will be allowed to discuss their decisions with family members before participating in this study. The wishes of the participant will be respected if they choose not to participate in the research. The research assistant or site principal investigator will provide a copy of the informed consent form and obtain written consent from the potential study participants. It will be highlighted to the participants that this study is voluntary, and their participation would not affect their clinical care or any benefits that they are entitled to.

Participants will be advised to retain a copy of the signed informed consent form for documentation, they will also receive a WhatsApp (Meta Platforms, Inc) message confirming their enrollment for this study, contact details, and basic information about study design.

### Sample Size and Power Calculation

Since this is a feasibility study, a formal sample size calculation is not required [34]. Our target is to recruit a minimum of 30 participants (with a maximum of 40), evenly divided into groups: persons living with diabetes and carers of persons living diabetes, with 15 participants in each group (with a maximum of 20). This approach is based on a previous study that shows a feasibility study requires 30 or more participants to estimate a parameter [35]. An arbitrary sample size of 30 would provide 0.75 (75%) power to detect a medium effect size of 0.5. Alternatively, a sample size of 40 would provide even greater power at 0.87 (87%).

### Intervention

#### Well Feet mHealth App

**Figure 1:**
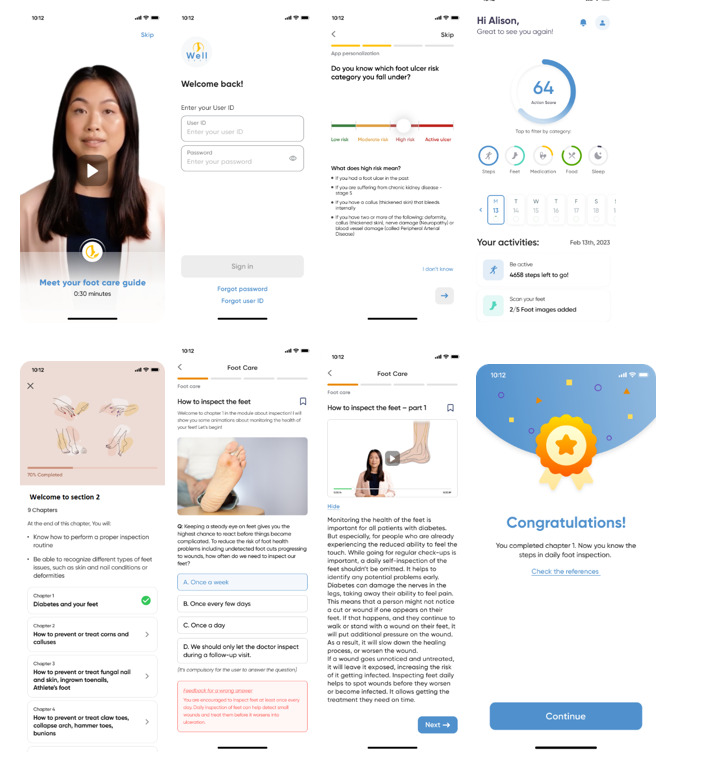
Selected screenshots from the Well Feet app.

Well Feet is a diabetes and foot care education mHealth developed by Nanyang Technological University. The technical building process of the app is outsourced to a university-appointed vendor while the content building process was carried out in-house. The app was made available to download both for iOS and Android. Screenshots of Well Feet’s main interface and features are shown in Figure 1.

#### Adaptive Learning Framework

Well Feet is a diabetes and foot care education app and was designed based on an adaptive learning framework to deliver educational modules and action-oriented conversations related to foot care through multilingual (English, Mandarin, and Malay) animations with a deep fake presenter. A total of 21 modules covering 4 themes: introduction, inspection, prevention, and wound care, have been developed by this study’s team based on a series of validated and evidenced-based sources including the International Working Group on the Diabetic Foot [36] guidelines as visualized in Textbox 1. The curriculum outline was consulted in 2 rounds in an asynchronous expert panel. Next, the scripts were developed with consideration of readability, cultural relevance, and local context, and were transformed into animations in an iterative design process comprising storyboarding, prototyping, production, and reviews. In total, 54 bite-sized and visually appealing animation videos ranging from 30 seconds to 2 minutes were produced for diabetes foot educational content. The content was prepared in English and translated into Mandarin and Malay. The animations are accompanied by adaptive learning quiz questions that allow us to assess knowledge gaps and trigger spaced learning conversations in case of incorrect answers. The system includes storing an in-depth click-level data which allows a vast analysis of learning and usage analytics.

**Overview of the 21 modules included in the Well Feet app.**

**Introduction**

- Categories of risk
- Amputation
- Diabetes and your feet: nerves, blood vessels, skin, joints

**Inspection**

- Visual inspection
- Foot conditions—skin and nails 1 (corns and calluses)
- Foot conditions—skin and nails 2 (fissures or fungal nail and skin [tinea pedis] or dystrophic nail or ingrown toenails)
- Foot conditions—deformities 1 (claw toes or hammer toes or bunions)
- Foot conditions—deformities 2 (Charcot foot)
- Foot conditions—peripheral neuropathy
- Foot conditions—peripheral vascular disease
- Other foot conditions commonly encountered by patients with diabetes
- Professional foot examination

**Prevention**

- Hygiene
- Diabetes-friendly footwear
- How to trim nails safely
- Skin care
- Simple exercises for the foot or calf
- Practices to avoid

**Wound care**

- Wound care—basic
- Diabetic foot ulcer
- Wound care—diabetic foot ulcer

Concurrent with curriculum development, the team was working on identifying relevant app features and conceptualizing app mechanics with instructional experience as a focus to connect user flow between features and align it with desirable behavior changes. In total, 3 levels of learning adaptations have been devised: (1) personalization of the content based on the role served, (2) personalization of the order of the modules based on the user’s risk stratification identified in the onboarding process, and (3) personalization of the content based on user’s knowledge level through knowledge assessment. This personalization forms the basis of learning framework in Well Feet. Additional features such a Frequently Asked Question chatbot, meal diary, feet diary, medication reminders steps, and sleep tracker are further added to enhance user experience. Finally, the app includes an automated notification system which in case of inactivity prompts the user to use the app.

#### Setting

Due to the nature of the Well Feet intervention being an mHealth app, participants are anticipated to engage with the intervention at their own discretion. Nevertheless, this study’s team will remind the participants at the start of this study to engage with Well Feet app throughout this study’s period.

### Data Collection

#### Overview

**Figure 2:**
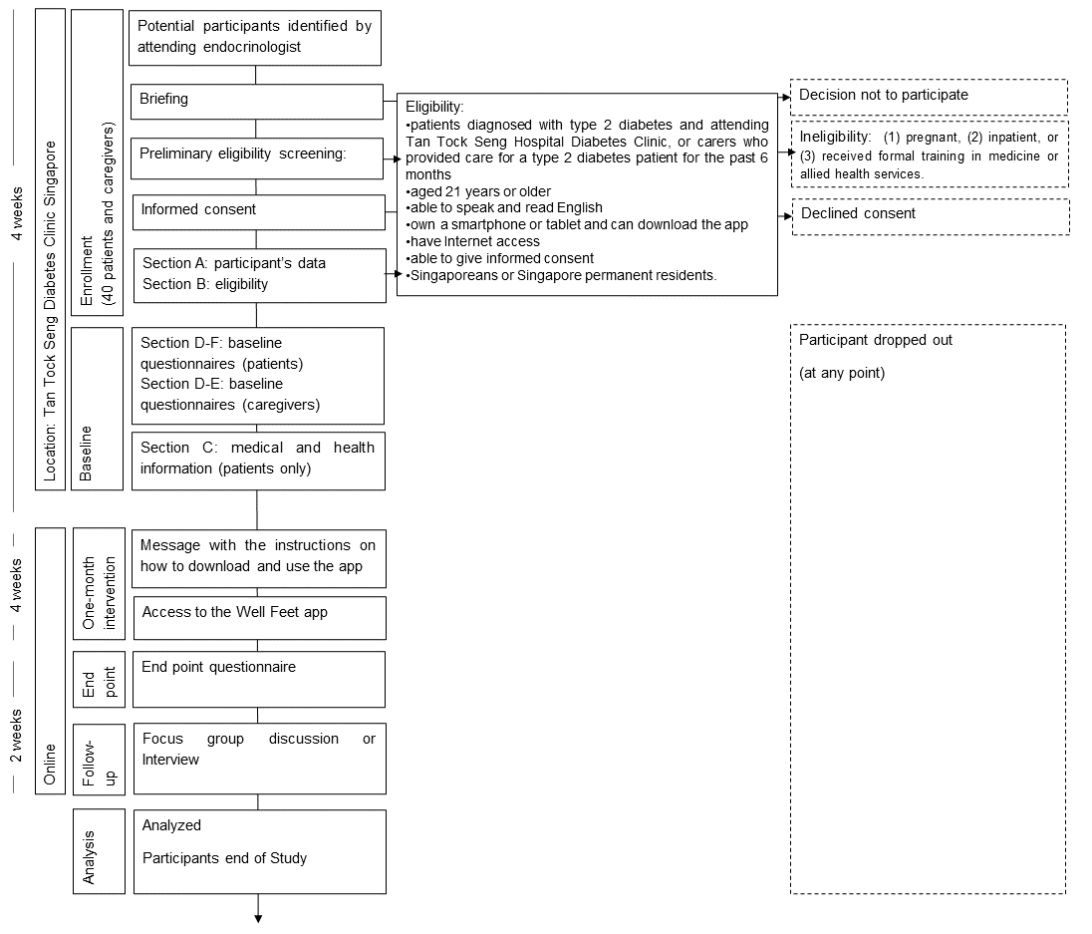
Participant flow through this study (CONSORT [Consolidated Standards of Reporting Trials]).

The participant flow through this study is illustrated in Figure 2. Consenting participants will be assigned with a study ID that will be used as their coded identifier throughout this study. Participants will receive a briefing regarding this study’s procedures and will answer a baseline questionnaire using a digital survey platform, REDCap (Research Electronic Data Capture, Vanderbilt University), accessed through a provided tablet. A study ID will be assigned to each participant to keep their answers to the questionnaire anonymous. A research assistant will assist with participants to complete the prescreening and enrollment sections, that is, for the participant's details and eligibility form. The clinical research assistant will access and extract medical records to complete the section with medical and health related information. Participants will complete sociodemographic information and questionnaire sections by themselves. The research assistant may assist the participants if help is needed. Additionally, the research assistant will access and extract medical records to complete the section with medical and health related information. Carers will go through the same recruitment and briefing flow as the patients. However, no medical record will be extracted for carers. Completion of the questionnaire is on a voluntary basis. If the participant requests to fill out the questionnaire at their own convenience, the research assistant will send them a link to the questionnaire through their preferred communication medium. The research assistant will also follow-up with a gentle reminder to complete the questionnaire in a case the participant did not fill it out.

The intervention will begin with the participants receiving the link to the app. The participants will be asked to use the app for up to 1 month. At the end of the period, they will be asked to fill out an end point questionnaire and will be invited to participate in focus group discussions or an interview, in case the participant cannot participate in focus group discussions, as a follow-up. To minimize attrition, prompts will be sent via the participants’ chosen mode of contact (eg, via email, telephone, SMS text message, or WhatsApp) to indicate it is time to complete the survey or participate in the focus group discussion.

#### Outcome Measures

This study’s outcomes of interest encompass methodological, procedural, and feasibility questions. Various factors are considered. Table 1 displays the primary outcomes including usability of the app and qualitative perspective on user experience. Multimedia Appendix 2 [37-43] displays secondary outcomes, including changes in foot care knowledge, self-management behaviors, and quality of life. Additionally, usage and learning analytics will be collected from the backend of the app.

**Table 1:** Primary outcomes.

Outcome measure			Measure description		Time frame
**Primary outcome**					
	Usability of a health app among patients and carers at the end of the trial (1 month)	A validated questionnaire, MAUQ^a^ [44], will be used to determine the usefulness and applicability of an app with a conversational agent or chatbot among patients and carers. The questionnaire consists of 3 subscales, which are ease of use (5 items), interface and satisfaction (7 items), and usefulness (6 items). Participants rate each of the items using a 7-point Likert scale ranging from 1 (strongly disagree) to 7 (strongly agree). The usability of the app is determined by the total and average of all statements—the higher the overall average, the better the usability of the app. However, if the average score is lower than 4, it means that the usability of the app is not good. The MAUQ exhibited strong psychometric characteristics, as evidenced by an overall Cronbach α value of .914 [44].		End-of-trial (1 month)	
	Qualitative perspective on a conversational agent or chatbot app usage experience among patients and carers at the end of the trial (1 month).	A focus group discussion among the app users will be carried out to collect feedback on the user experience at the end of the trial. Qualitative data on app usability, app applicability, app relevance, and user feedback will be retrieved from the focus group discussion.		End-of-trial (1 month)	

^a^MAUQ: mHealth (mobile health) App Usability Questionnaire.

### Data Management

All data will be handled according to patient Nanyang Technological University data protection and privacy policy [45] as well as National Healthcare Group, Tan Tock Seng Hospital data protection and privacy policy [46] with all participants assigned a study ID to deidentify data and any personal information about participants will be stored separately from deidentified data. Data collected via digital survey platform, REDCap will be stored on secure servers at Center for Population Health Sciences, Lee Kong Chian School of Medicine, Nanyang Technological University. The data gathered through the app will be stored in a university-approved cloud server as a multiencrypted database with authorization granted for principal investigators and study team members to prevent unauthorized persons from accessing the data. All other physical forms will be stored in a locked filing cabinet in the clinical site and will be accessible only to the site-principal investigator and appointed research assistant. User’s interaction within the Well Feet app will be monitored to identify participants’ signaling their need for more extensive support.

### Ethical Considerations

This study was approved by the National Healthcare Group Domain Specific Review Board (2022/00614) and the Nanyang Technological University institutional review board (IRB-2023-157), ensuring that the welfare and rights of all participants is protected in this study. This study will be conducted in accordance to the Helsinki code of ethics ensuring that patients’ confidentiality, integrity, and vulnerability are kept throughout their participation. Informed consent will be collected to ensure participants are aware of this study’s procedure and their rights to withdraw from this study without the need to give any reason. Participants will be provided with contact information within the informed consent form should they have any concern regarding the conduct of the feasibility study.

### Analysis

#### Quantitative Analyses

The questionnaires answered by the participants will be summarized using descriptive statistics, and the data will be presented as frequencies, percentages for categorical variables and means, SDs, medians, and IQRs for continuous variables depending on the distribution of the variable. Descriptive statistics will also be used to assess measures of app usage. Differences between groups will be analyzed using chi-squared or Fisher exact test for nominal data and changes within the groups over time (pre-post) will be analyzed using the Wilcoxon signed rank test. R (R Core Team) and Stata (StataCorp) will be used for all analyses.

#### Qualitative Analyses

All focus group discussions will be recorded on a digital teleconferencing platform (ie, Zoom; Zoom Video Communications, Inc), which will then be transcribed verbatim. Any identifiers in the transcriptions will be removed. Thematic analysis will be used to identify participants’ experience, overall acceptance, and feedback. The analysis of the responses will start immediately following the first focus group and transcription. Initial codes will be generated, then collated into themes to identify the key patterns and key themes of the focus group discussions using the procedures suggested by Clarke and Braun [47].

### Dissemination

This study’s findings will be published in an open access journal and via national and international conference presentations.

## Results

This study was funded in October 2019 and received ethics approval in February 2023. Recruitment of this study was done in July 2023 with 40 participants enrolled. Well Feet app is expected to be published at Google Play and iOS testflight at the end of August 2023, followed by a month-long intervention period. Analysis of results is expected to be completed by October 2023, and trial results are expected to be published in spring 2024.

## Discussion

### Principal Findings

This study is designed with the aim to evaluate the feasibility of Well Feet, a mobile health app designed for people living with diabetes and at risk of DFU. This study will provide insights on Well Feet’s added values, potential benefits, and challenges of its use among patients and carers. Through a combination of quantitative evaluation using the validated MAUQ questionnaire and qualitative insight from focus group discussions, this study will provide a perspective on the feasibility of Well Feet particularly on how the app facilitates patients and carers engagement in diabetes foot health management. Additionally, analytics derived from Well Feet usage data will provide a clearer picture on user interaction with features of the app.

Studies ranging from early detection and prevention to self-monitoring apps collectively highlight the potential of mHealth in improving patient outcomes, and addressing challenges in diabetes care [48]. We are expecting that similar to other studies a difference in knowledge pretest and posttest knowledge score will be observed [49,50] and that the app will be perceived as valuable [22,24,25,27,28,51]. However, as the learning curriculum included in Well Feet app is much more comprehensive than in other solutions and encompasses multilevel tailoring mechanisms such as notifications for learning reinforcement and adaptive learning pathway for knowledge gaps identification, we are expecting that the foot care knowledge and behavior results will differ from other studies. The qualitative analysis will additionally help to identify specific behavioral changes among study participants going beyond observing frequency of foot checking [20].

Furthermore, in this study we are aiming at in app exploring usage patterns among the participants, especially those who experienced knowledge or behavior change. Ploderer et al [51] has observed 3 usage patterns when evaluating a wound care tracking app: continuous, temporary, and failing usage. In our study, it is expected that some barriers to usage identified by Ploderer et al [51] will overlap, especially the ones leading to temporary usage such as work commitments and health disruptions. As the sample of participants will vary across different risks of developing DFU and will include low and moderate risk patients, the frustration with lack of healing progress might not arise. The usage and learning analytics of click-level data should allow us to further describe exact app behaviors that constitute each group of users. Usage patterns will be juxtaposed with knowledge and behavior questionnaires’ results to further reveal engagement patterns and factors influencing the success of foot care education.

### Strengths and Limitations

This study comes with a set of strengths and limitations. A structured approach described in this protocol will be used to assess the practicality and viability of a newly developed adaptive diabetes foot health education, Well Feet, before a full-scale development and deployment. The utilization of both quantitative and qualitative measures enhances the depth and breadth of insights gained from this evaluation. By performing a feasibility study, the research team will be able to identify technical, operational, and logistical challenges early in the process, thus potentially saving time and resources when a larger randomized controlled trial is conducted.

However, there are several limitations worth noting. The small cohort of study participants recruited from a single tertiary hospital limits the generalizability of findings. A follow-up multicenter randomized controlled trial is warranted to predict a proper intervention effect. Second, while only patients who owned mobile devices were recruited, no formal assessment of digital literacy was performed during the recruitment process. As smartphone ownership may not be an accurate indicator of proficiency in using basic phone features [52], patient’s engagement with Well Feet app may be impacted by limited digital literacy.

### Conclusions

Patient-facing digital health technologies such as mHealth apps promise to support self-management of various health conditions, including diabetes and associated risk factors. Such feasibility research, especially when it encompasses user-centered design and evaluation, contributes to health care services transformation aimed at equitability, patient empowerment, and shared decision-making. Gaining growing attention from researchers [53] and patients [15], mHealth interventions pave the road for future health care where digital solutions, including health education, are seamlessly integrated into routine prevention and care. This feasibility study is a step in embracing technological innovations which aim to optimize advancing a DFU prevention and management and care delivery.

## Appendix

Multimedia Appendix 1 Informed consent form.

Multimedia Appendix 2 Secondary outcomes.

## Abbreviations

CONSORT: Consolidated Standards of Reporting Trials
DFU: diabetic foot ulcer
mHealth: mobile health
REDCap: Research Electronic Data Capture

## Data Availability

Deidentified interview data are available on request. Researchers who wish to obtain further details may contact the corresponding author with a methodologically sound proposal.
