# Supplementary material for: Empowering Foot Care Literacy Among People Living With Diabetes and Their Carers With an mHealth App: Protocol for a Feasibility Study
Source: JMIR Res Protoc. 2023 Nov 21;12:e52036. doi: 10.2196/52036 (PMC10698655; doi:10.2196/52036)
Supplement: Multimedia Appendix 1 [file resprot_v12i1e52036_app1.docx]

# Multimedia Appendix 1


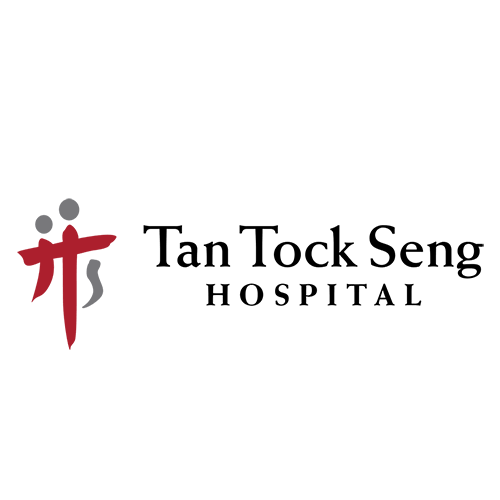
**
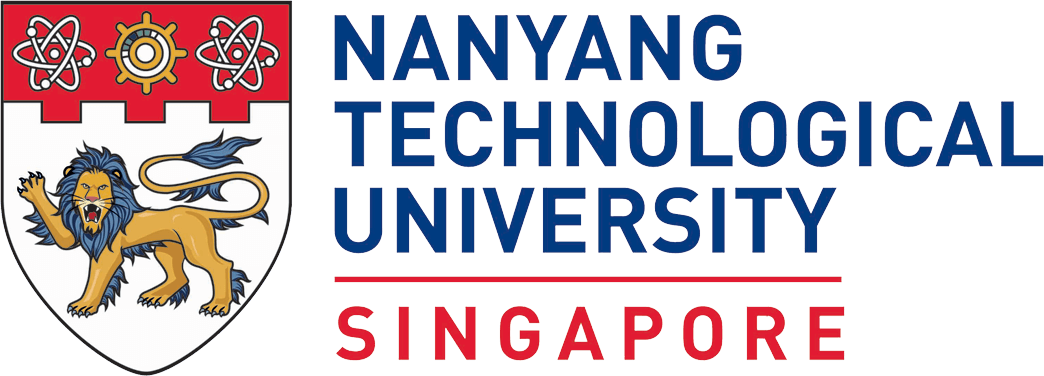

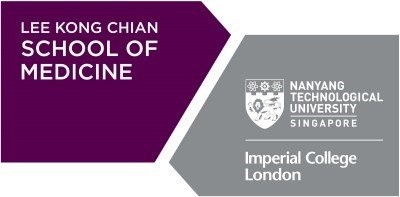
**

**INFORMED CONSENT FORM FOR PATIENT AND CARER**

| **1. Study Information** |
| --- |
| **Protocol Title:** |
| A Feasibility Study of an App for Empowering Foot Care Literacy among People with Diabetes and their Carers |
| **Principal Investigator & Contact Details:** |
| TTSH Site Principal Investigator: Dr Liew Huiling  Consultant  Endocrinology Department  Tan Tock Seng Hospital  11 Jln Tan Tock Seng, Singapore 308433  Email: huiling_liew@ttsh.com.sg  Contact No: +65 6357 7000  NTU Site Principal Investigator: Professor Josip Car  Director of Centre for Population Health Sciences  Lee Kong Chian School of Medicine  Nanyang Technological University  11 Mandalay Rd, Singapore 308232  Email: josip.car@ntu.edu.sg  Contact No: +65 9059 3462, 85729049 |
| **Study Sponsor:** |
| National Medical Research Council, Health Services Research Grant (HSRG-DB17Nov002) |
| **2. Purpose of the Research Study**  The aim of this study is to assess the feasibility and acceptability of a conversational agent app, ‘Well Feet’, to provide education and self-management support for diabetes foot and foot ulcers care among people with diabetes and their carers.  This study will recruit a total of 30 participants (15 patients and 15 patients with carers) from Tan Tock Seng Hospital (TTSH) Diabetes Clinic over a period of 2 months*.* |
| **3. Invitation to Participate in This Study**  You are being invited to participate because:  For patient:   1. You have type 2 diabetes and are currently attending TTSH Diabetes Clinic as an outpatient; 2. You are 21 years old or above; 3. You can speak and read English or Mandarin or Bahasa Melayu; 4. You own a smartphone or tablet; 5. You can download the study app; 6. You have internet access; 7. You are a Singaporean or Singapore Permanent Resident; 8. You are not pregnant; 9. You have not received any formal training in medicine or other health-related qualifications; 10. You can give informed consent.   For carer:   1. You are an informal caregiver for a patient with type 2 diabetes currently attending TTSH Diabetes Clinic as an outpatient; 2. You are 21 years old or above; 3. You can speak and read English or Mandarin or Bahasa Melayu; 4. You own a smartphone or tablet; 5. You can download the study app; 6. You have internet access; 7. You have not received any formal training in medicine or other health-related qualifications; 8. You can give informed consent.   Nevertheless, taking part in this research study is entirely your choice. Please read all of the following information carefully. Feel free to ask research team to explain any words, terms, or sections that are unclear to you. Please do not sign this consent form unless you understand the information in it and have had your questions answered to your satisfaction. You will be given a copy of the signed form. You should keep your copy for your records. |
| **4. What Procedures will be Followed in This Study** |
| If you (patient) agree to participate in this study, you will be first asked to nominate a carer to participate in the study. However, this is optional. So, you can still participate in the study without a carer. Your participation in the study will last for up to two months.  Following this, both sets of participants (patients and carers) will:   1. complete 2 visits to the Tan Tock Seng Hospital (TTSH) Diabetes Clinic during the study. 2. Download the ‘Well Feet’ app onto the mobile phone and use it regularly for a period of 1 month, i.e. at least 4 times in a week, each session 5 to 10 minutes. 3. Engage with the app by reading foot care educational modules and complete quizzes to assess foot care knowledge*. 4. Complete a pre-test survey at baseline, which takes approximately 30 minutes. Complete a post-test survey 1 month after baseline, which takes approximately 30 minutes.  If you request to fill out the questionnaire and download the app at your own convenience, CRC will send you a link to the questionnaire through your preferred communication medium. CRC may also follow up with a gentle reminder to complete the questionnaire. 5. Take part in an online focus group discussion (FGD) within 1 month after the post-test survey to share your experiences using the app. If participating in the FGD makes you to feel uncomfortable, you may choose to attend one-to-one interview session. Both FGD and interview will be conducted through a digital teleconference platform, Zoom. Each FGD session will take approximately 60 minutes to complete while each interview session will take approximately 30 minutes to complete.   *There is no requirement to complete all the educational modules or to complete all the quiz assessments.  Additionally, for patients, this study involves access to and extraction of data from your medical records at baseline. For this study, the following information will be extracted:   1. Month and year of birth (MM/YYYY) 2. Gender 3. Year of diagnosis 4. Complications (e.g., neuropathy, peripheral vascular disease, etc.). 5. Family history of diabetes 6. Smoking status 7. Alcohol consumption 8. Diagnosed conditions (condition, year of onset, current problem – yes/no) 9. Current use of prescribed medications (name of medication, dosage, dosage frequency) 10. Current use of complementary medications   For carers, this study will not retrieve any medical records.  Incidental findings are findings that have potential health or reproductive importance to research participants. As there is no new investigation in this study, there will be no incidental findings arising in this research. |
| **5. Your Responsibilities in This Study** |
| If you agree to participate in this study, you will follow the procedures described in Section 4. Additionally, you should follow the advice given to you by the study team throughout the study period. |
| **6. What Is Not Standard Care or is Experimental in This Study** |
| The procedures described in Section 4 are conducted for research purposes and are not part of your routine care received at TTSH Diabetes Clinic. |
| **7. Possible Risks and Side Effects**  The risks in participating in this study are minimal and involve no medical risks to you. However, you may find the questionnaire or FGD/interview session too lengthy. In the event you feel fatigued or distracted, you may take breaks as needed or terminate the survey at any timepoint. Additionally, significant psychological risk is not anticipated in this study, but there is a small possibility that the questionnaire or FGD/interview session involved in this study may arouse some emotional response to events such as previous amputations and disputes with caregivers, or distress symptoms such as crying. Should this happen, you are advised to take breaks or you may withdraw from the study at any time. For the questionnaire survey, all questions are compulsory. However, if you have issues addressing certain questions, you are allowed to withdraw from the study, even if you are able to answer the majority of the questions. |
|  |
| **8. Possible Benefits from Participating in the Study** |
| If you participate in this study, you may reasonably expect to benefit from using the app in the following way: it may increase your foot care knowledge and provide you with foot care strategies and tips. Moreover, the findings from this study will be used to inform a full intervention trial, and researchers may use the findings to aid in the future development of digital health interventions that cater to people with diabetes and their carers. |
|  |
| **9. Alternatives to Participation** |
| If you choose not to participate in this study, you will continue to receive the standard care for your condition at TTSH Diabetes Clinic. By standard care, it refers to the routine medical care offered by healthcare professionals for your condition.  If you are a carer and choose not to participate in this study, you will still be able to play your role as a carer for your patient. Your choice to not participate will not affect your or your patient’s medical benefits and entitlements. |
| **10. Costs & Payments if Participating in the Study** |
| There is no cost incurred to you for your participation in this study.  You will be reimbursed for your time and transportation costs as follows:   - The first payment of $30 will be given after the completion of the pre-test survey (Visit 1), and a second payment of $40 will be given after the completion of the post-test survey (Visit 2). The third payment of $80 will be given after attendance of the FGD/interview session. Payments will be made in the form of vouchers (e.g., NTUC FairPrice vouchers). - Participants who complete the pre-test survey but choose to withdraw partway through the study will not receive the second and subsequent payments. - Participants who have consented, but do not complete the pre-test surveys for any reason, are considered automatically withdrawn from the study and will receive no payment. |
| 1. **Voluntary Participation** |
| Your participation in this study is voluntary and you may stop participating at any time. Your decision to not take part in this study or to terminate your participation will not affect your routine medical care at TTSH Diabetes Clinic or any benefits to which you are entitled. If you choose to terminate your participation, you will be required to notify us so we can proceed with an orderly termination. This can be done by contacting one of the Principal Investigators. Contact information of the Principal Investigators can be found in Section 14.  Upon study termination, the clinical research assistant can help you to uninstall the app from your device. However, the data that has been collected until the time of your withdrawal will be kept and used for data analysis. The reason is to enable a complete and comprehensive evaluation of the study.  Your doctor, the Principal Investigators and/or the Sponsor of this study may stop your participation in the study at any time if they decide that it is in your best interests. They may also stop your participation if you do not follow instructions required to complete the study adequately. If you have other medical problems or side effects, the doctor and/or nurse will decide if you may continue to participate in the study.  In the event of any new information becoming available that may be relevant to your willingness to continue in this study, you *(or your legally acceptable representative, if relevant*) will be informed in a timely manner by the Principal Investigator or his/her representative.   1. **Compensation for Injury**   If you follow the directions of the doctors in charge of this study and you are physically injured due to the procedures involved in this study, Tan Tock Seng Hospital will pay the medical expenses for the treatment of that injury.  Payment for the management of the normally expected consequences of your treatment will not be provided by Tan Tock Seng Hospital. In signing this consent form, you will **not** waive any of your legal rights or release the parties involved in this study from liability for negligence. |
| **13. Confidentiality of Personal Data** |
| Your participation in this study will involve the collection of “Personal Data”. “Personal Data” means data about you which makes you identifiable (i) from such data or (ii) from that data and other information which an organization has or is likely to have access. This includes medical records regarding conditions, medications, investigations, and treatment history.  Information and “Personal Data” collected for this study will be kept confidential. Your medical records, to the extent of the applicable laws and regulations, will not be made publicly available.  However, the NHG Domain Specific Review Board and the Ministry of Health will be granted direct access to your original medical records to check study procedures and data, without making any of your information public. By signing the Informed Consent Form attached, you (*or your legally acceptable representative, if relevant*) are authorising (i) the collection, access, use and storage of your “Personal Data”, and (ii) the disclosure to authorised service providers and relevant third parties.  All collected data will be kept confidential and remain as a shared property of TTSH and NTU and will be used only for research purposes. In the event of any publication regarding this study, your identity will remain confidential.  Research arising in the future, based on your “Personal Data”, will be subject to review by the relevant institutional review board. By participating in this research study, you are confirming that you have read, understood and consent to Tan Tock Seng Hospital Data Protection Privacy Notice available at <https://www.ttsh.com.sg/About-TTSH/TTSH-Community-Fund/Pages/Data-Protection-Privacy-Notice.aspx> and Nanyang Technological University Privacy Statement available at https://www.ntu.edu.sg/footer/ntu-privacy-statement#Content_C087_Col00. |
| **14. Who to Contact if You Have Questions**  If you have questions about this research study, you may contact the Principal Investigator,  TTSH Site Principal Investigator: Dr Liew Huiling  Consultant  Endocrinology Department  Tan Tock Seng Hospital  11 Jln Tan Tock Seng, Singapore 308433  Email: [huiling_liew@ttsh.com.sg](mailto:huiling_liew@ttsh.com.sg)  Contact No: +65 6357 7000  NTU Site Principal Investigator: Professor Josip Car  Director of Centre for Population Health Sciences  Lee Kong Chian School of Medicine  Nanyang Technological University  11 Mandalay Rd, Singapore 308232  Email: [josip.car@ntu.edu.sg](mailto:#osip.car@ntu.edu.sg)  Contact No: +65 9059 3462, 8672 9049  The research team is available in case of any queries or technical difficulties etc. The study team may also contact the participant sharing links to questionnaires or link to the app, and arranging the endpoint questionnaire visit, interview or FGD.  In case of any injuries during the course of this study, you may contact the above-named Principal Investigators  The study has been reviewed by the NHG Domain Specific Review Board (the central ethics committee) for ethics approval.  If you want an independent opinion to discuss problems and questions, obtain information and offer inputs on your rights as a research subject, you may contact the NHG Domain Specific Review Board Secretariat at 6471-3266. You can also find more information about participating in clinical research, the NHG Domain Specific Review Board and its review processes at [www.research.nhg.com.sg](http://www.research.nhg.com.sg).  If you have any complaints or feedback about this research study, you may contact the Site-Principal Investigator or the NHG Domain Specific Review Board Secretariat. |

#### CONSENT FORM

| **Protocol Title:** |
| --- |
| A Feasibility Study of an App for Empowering Foot Care Literacy among People with Diabetes and their Carers |
| **Principal Investigator & Contact Details:**  TTSH Site Principal Investigator: Dr Liew Huiling  Consultant  Endocrinology department  Tan Tock Seng Hospital  Email: huiling_liew@ttsh.com.sg  Contact No: +65 6357 7000  NTU Site Principal Investigator: Professor Josip Car  Director of Centre for Population Health Sciences  Lee Kong Chian School of Medicine  Nanyang Technological University  Email: josip.car@ntu.edu.sg  Contact No: +65 6904 7005 |
|  |

I voluntarily consent to take part in this research study. I have fully discussed and understood the purpose and procedures of this study. This study has been explained to me in a language that I understand. I have been given enough time to ask any questions that I have about the study, and all my questions have been answered to my satisfaction. I have also been informed and understood the alternative treatments or procedures available and their possible benefits and risks.

By participating in this research study, I confirm that I have read, understood and consent to the Tan Tock Seng Hospital Data Protection Privacy Notice and Nanyang Technological University Privacy Statement.

Name of Participant Signature Date

**Investigator Statement**

I, the undersigned, certify that I explained the study to the participant and to the best of my knowledge the participant signing this informed consent form clearly understands the nature, risks and benefits of his / her participation in the study.

________________________ _________________ ________________

Name of Investigator / Signature Date

Person administering consent
