# Supplementary material for: Empowering Foot Care Literacy Among People Living With Diabetes and Their Carers With an mHealth App: Protocol for a Feasibility Study
Source: JMIR Res Protoc. 2023 Nov 21;12:e52036. doi: 10.2196/52036 (PMC10698655; doi:10.2196/52036)
Supplement: Multimedia Appendix 2 [file resprot_v12i1e52036_app2.docx]

**Table S1.** Secondary outcomes.

| Outcome measure | | Measure description | | | Time frame |
| --- | --- | --- | --- | --- | --- |
| **Secondary outcomes** | | | | | |
|  | Changes in foot care knowledge among patients and carers at baseline and end-of-trial (1 month). | | A validated questionnaire, Foot Care Knowledge Questionnaire [39] will be used to evaluate changes in patient's and carer's knowledge on foot care from baseline to the end-of-trial. The questionnaire consists of 15 items to be rated true or false and the score will be presented in terms of percentage of correct answers. A higher percentage of correct answers means higher level of foot care knowledge. The Foot Care Knowledge Questionnaire has been widely employed as a quantitative assessment tool for evaluating diabetic patients' knowledge regarding foot care, and is proven as a valid measurement tool [40]. | Baseline and end-of-trial (1 month) | |
|  | Changes in foot care related self-management behavior among patients at baseline and end-of-trial (1 month). | | A validated questionnaire, Nottingham Assessment of Functional Footcare [41], will be used to assess changes in patient's level of foot care related self-management behavior from baseline to the end-of-trial. This questionnaire consists of 29 items on a 5-point Likert scale. The total number of scores will be added up and we will multiply the score by 1.115 to obtain the final score. A higher score means better self-management care. The Nottingham Assessment of Functional Footcare has demonstrated reliability and validity in measuring foot self-care practices (behaviors), exhibiting a strong test-retest reliability coefficient of 0.87 [42]. | Baseline and end-of-trial (1 month) | |
|  | Changes in carer's quality of life at the baseline and end-of-trial. | | A validated questionnaire, Brief Assessment Scale for Caregivers [43], will be used to determine changes in carer's quality of life and level of burden from caring for a person with diabetes. This questionnaire consists of 14 items clustered into 5 factors. The mean Brief Assessment Scale for Caregivers score is computed by summing up the non-not applicable items in each factor, then dividing by the number of items that were not missing. This gave a score scaled from 0 to 3, with a higher score indicating better caregiver outcomes. The Brief Assessment Scale for Caregivers displayed a favorable internal reliability of 0.70 and validated its construct through correlation assessments with instruments that gauge different aspects related to caregiver burden [43]. | Baseline and end-of-trial (1 month) | |
|  | Login frequency in the conversational agent or chatbot app among patients and carers at the end of the trial (1 month). | | Data on login frequency will be retrieved from the backend of the app at the end-of-trial. | End-of-trial (1 month) | |
|  | Time spent on the app in the conversational agent or chatbot app among patients and carers at the end of the trial (1 month). | | Data on total number of minutes spent on the app will be retrieved from the backend of the app at the end-of-trial. | End-of-trial (1 month) | |
|  | Number of modules accessed in the conversational agent or chatbot app among patients and carers at the end of the trial (1 month). | | Data on total number of education module accessed will be retrieved from the backend of the app at the end-of-trial. | End-of-trial (1 month) | |
|  | Number of modules completed in the conversational agent or chatbot app among patients and carers at the end of the trial (1 month). | | Data on number of education module completed will be retrieved from the backend of the app at the end-of-trial. | End-of-trial (1 month) | |
|  | Module quizzes’ scores in the conversational agent or chatbot app among patients and carers at the end of the trial (1 month). | | At the end of each module, users will be directed to a module quiz. Data on total scores for the module quizzes will be collected from the backend of the app at the end-of-trial. | End-of-trial (1 month) | |
|  | Module rating in the conversational agent or chatbot app among patients and carers at the end of the trial (1 month). | | At the end of each module, users will be prompted to rate their learning experience using a 5-point Likert scale. Data of the ratings on each of the modules will be collected from the backend of the app at the end-of-trial. | End-of-trial (1 month) | |
|  | Overall app rating for the conversational agent or chatbot app among patients and carers at the end of the trial (1 month). | | Upon completion of all learning modules, users will be prompted to rate their overall experience of using the app using a 5-point Likert scale. Data of the overall experience of app rating will be retrieved from the backend of the app at the end-of-trial. | End-of-trial (1 month) | |
|  | Usability of a conversational agent or chatbot among patients and carers at the end of the trial (1 month). | | A validated questionnaire, Chatbot Usability Questionnaire [44], will be used to determine the usefulness and applicability of a conversational agent or chatbot among patients and carers at the end of the trial. This questionnaire consists of 16 items on a 5-point Likert scale. The mean score will be calculated using Chatbot Usability Questionnaire calculator available on the Ulster University website. This higher mean score means better chatbot usability. The Chatbot Usability Questionnaire has been tested in a variety of populations, ranging from the general public to health workers, and has proven to be a reliable and validated instrument [45]. | End-of-trial (1 month) | |
